# P16 Updating paediatric antimicrobial guidelines: lessons learnt

**DOI:** 10.1093/jacamr/dlae217.020

**Published:** 2025-01-23

**Authors:** Marianna Przybylska, Carol Philips, Laura Jones, Pota Kalima, Naomi Henderson, Simon Dewar

**Affiliations:** NHS Lothian, Edinburgh, UK; NHS Lothian, Edinburgh, UK; NHS Lothian, Edinburgh, UK; NHS Lothian, Edinburgh, UK; NHS Lothian, Edinburgh, UK; NHS Lothian, Edinburgh, UK

## Abstract

**Background:**

Regularly reviewing local guidelines is essential to ensure that clinical practice aligns with national standards and incorporates the latest evidence-based recommendations. To optimize patient care and strengthen antimicrobial stewardship, antimicrobial guidelines need to account for evolving microbial resistance patterns, including regional trends; consider the latest evidence on drug efficacy, side effects and new antimicrobial options; account for changing disease patterns; and provide specific guidance for at risk populations that may require tailored treatment advice.

**Objectives:**

The primary objective was to update our local paediatric antimicrobial guidelines to ensure alignment with the UK Paediatric Antimicrobial Stewardship (UKPAS) Network recommendations.

**Methods:**

A multidisciplinary guideline review committee was established, consisting of consultant microbiologists, a consultant in paediatric infectious diseases, a clinical development fellow and a pharmacist. Local antimicrobial guidelines were reviewed and updated, aligning them with UKPAS recommendations. The revised guidelines were subsequently submitted to the Antimicrobial Committee for further review and approval.

**Results:**

The review process highlighted the importance of regularly updating guidelines to reflect emerging evidence, new drug recommendation (e.g. from MHRA), and evolving microbial resistance patterns. It also provided an opportunity to reassess and strengthen local antimicrobial stewardship initiatives. Key changes included recommendations aimed at reducing unnecessary antibiotic use e.g. shortening antibiotic course durations where clinically appropriate, enhancing antimicrobial education for clinicians, and improving the dissemination of information to staff. IV to oral switch guidance was updated, adopting recent UKSHA guidance. Additionally, a review of current practices revealed the potential need for further education to bridge gaps between existing practices and new recommendations, ensuring successful implementation and adherence to the updated guidelines. Interdisciplinary collaboration was crucial in achieving a comprehensive and balanced update of the guidelines. Involvement of the pharmacy team ensured considerations related to drug availability and resource management were considered. This helped ensure the proposed changes were feasible.

**Conclusions:**

Interdisciplinary approach to updating local antimicrobial guidelines not only ensures clinical practice is aligned with national standards but also provides a valuable opportunity to maintain effective antimicrobial stewardship, optimize resource management, and improve staff education and engagement with the guidelines. There are ongoing challenges in incorporating increased antibiotic dosing guidelines in paediatrics (to comply with EUCAST reporting of ‘I’) and paediatric-specific guidance for MHRA warnings (e.g. fluoroquinolones) to local policies. Through ongoing dialogue and guidance from national initiatives like UKPAS, these challenges are being met.

